# Advanced hepatocellular carcinoma with MET-amplified contained excellent response to crizotinib: a case report

**DOI:** 10.3389/fonc.2023.1196211

**Published:** 2023-08-16

**Authors:** Yangjun Gu, Min Xiao, Zhitao Chen, Qiyong Li

**Affiliations:** Department of Hepatobiliary and Pancreatic Surgery, Shulan (Hangzhou) Hospital Affiliated to Zhejiang Shuren University Shulan International Medical College, Hangzhou, China

**Keywords:** hepatocellular carcinoma, next-generation sequencing, MET, targeted therapy, comprehensive cancer therapy

## Abstract

**Introduction:**

Hepatocellular carcinoma (HCC) is one of the most lethal cancers worldwide. Several novel therapeutic strategies have been developed to prolong the survival of patients with advanced HCC. However, therapeutic decision-making biomarkers owing to the extensive heterogeneity of HCC. Next-generation sequencing (NGS) is generally used in treatment decisions to help patients benefit from genome-directed targeting.

**Case presentation:**

A 56 year-old male with type-B hepatitis for more than 20 years was admitted to our department and underwent laparoscopic left lateral hepatic lobectomy for hepatocellular carcinoma. Unfortunately, the tumor recurred 1 year later. Despite multiple treatments, the tumor continued to progress and invaded the patient’s 5th thoracic vertebras, leading to hypoesthesia and hypokinesia below the nipple line plane 2 years later. NGS revealed MET amplification, and crizotinib, an inhibitor of MET, was recommended. After administration for a month, tumor marker levels decreased, and the tumor shrunk. The patient has remained in remission since that time.

**Conclusions:**

We report that a patient with high MET amplification benefited from its inhibitor, which was recommended by NGS. This indicates the potential clinical decision support value of NGS and the satisfactory effect of MET inhibitors.

## Introduction

Hepatocellular carcinoma (HCC) is an aggressive malignant tumor that affects more than 600,000 people worldwide (1). Because of the high incidence of hepatitis virus infection, China has been considered as a “large country for liver cancer” (2). With advancing methods, such as new targeting concepts and immune therapies, clinicians not only significantly prolong the survival of patients but also improve their quality of life. However, the response rates to these treatments were unsatisfactory. The classic targeting concept sorafenib, for example, which was recommended as the first-line treatment therapy by almost all guidelines, was reported to benefit only about 40% of patients due to genetic heterogeneity and other reasons (3). The checkpoint inhibition immunotherapy nivolumab was reported a six-month survival rate of 83% and a nine-month survival rate of 74% with a 15% overall objective response rate (ORR) (4). Novel therapies combining immune checkpoint inhibitors with anti-angiogenic agents or tyrosine kinase inhibitors have demonstrated exciting outcomes. Atezolizumab plus bevacizumab reached 67.2% overall survival at 12 months and 6.8 months median progression-free survival, which is much better than tyrosine kinase inhibitor alone (5). However, it is disappointing that lenvatinib plus pembrolizumab for the treatment of advanced HCC (NCT03713593) is not better than lenvatinib alone. Although these therapies have persistent progression, primary hepatic cancer remains the most lethal tumor worldwide with a 5-year survival rate of 18% (3).

Due to the genomic instability of carcinoma, it presents genetic and phenotypic variations within and between tumors (6). Correspondently, patients are not only in different tumor performance but also in different reactions with the same treatment. The side effects of treatment, such as diarrhea, hypertension, and bleeding, also limit its efficacy. For instance, the incidences of hypertension, diarrhea, decreased appetite, and decreased weight for lenvatinib were 42%, 39%, 34%, and 31%, respectively. Approximately 75% of patients experience grade 3 or higher treatment-emergent adverse events, leading to drug interruption (32.2%), dose reduction (38.1%), and drug withdrawal (7.2%) (7). Thus, considering the response and tolerance rates, some patients still could not receive appropriate treatment or had prolonged survival.

Here, we present a case of HCC that achieved a satisfactory partial response to crizotinib. The fundamental information, process of diagnosis and treatment, genetic test report, and evaluation of the treatment effectiveness of the patient were established in this article. We expect this study to provide new ideas for the diagnosis and treatment of advanced HCC.

## Case presentation

**Figure 1:**
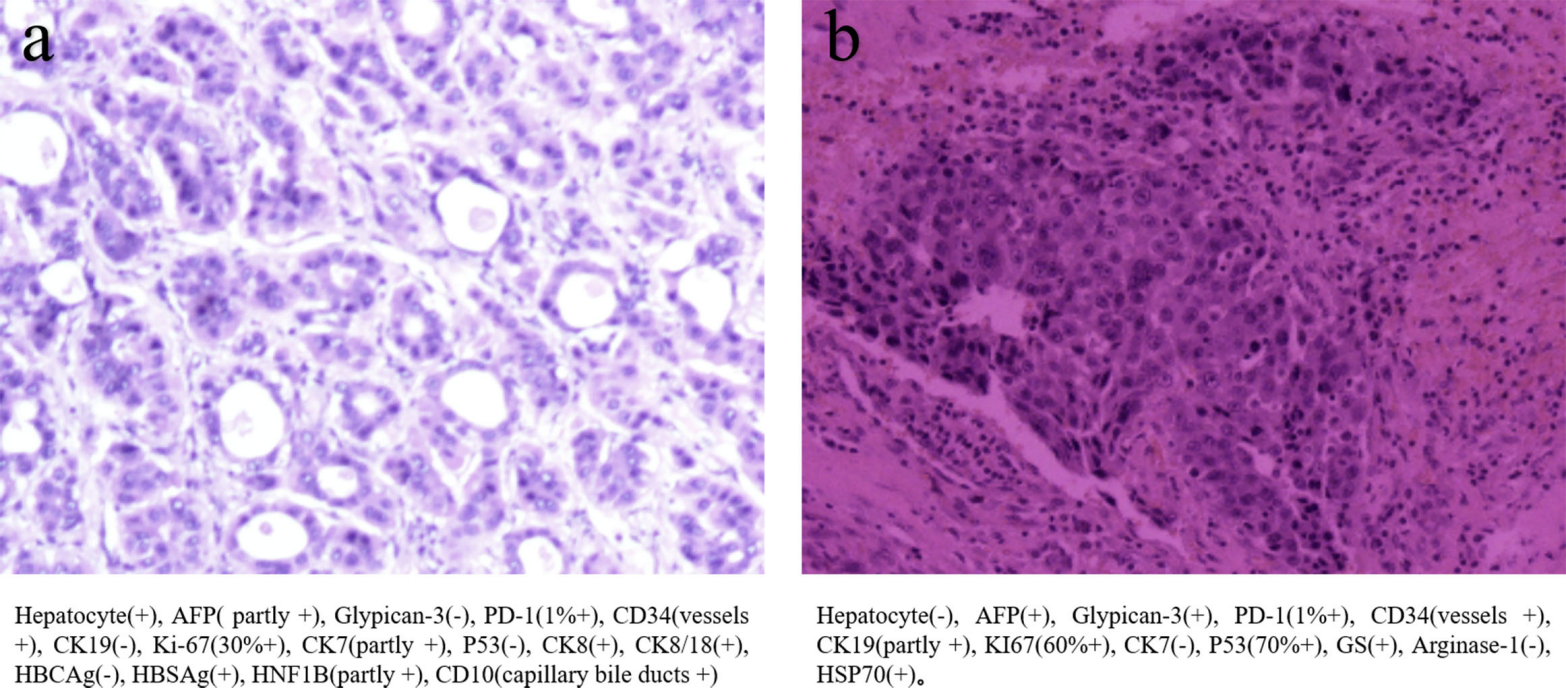
**(A)** The primary biopsy showed moderately to highly differentiated hepatocellular carcinoma **(B)** The biopsy of 5th thoracic vertebras metastasis, which showed several immunohistochemical difference between before and after.

A 56 year-old male was admitted to our department for liver mass. Because of type B hepatitis for more than 20 years and inappropriate and discontinuous anti-viral therapy, he was diagnosed with slight liver cirrhosis several years before admission. There were few positive findings on physical examination. Based on the elevated serum alpha-fetoprotein level (51 ng/ml) and typical imaging findings, the patient was diagnosed with hepatocellular carcinoma with a history of hepatitis. The patient underwent laparoscopic left lateral hepatic lobectomy on 23 May 2019. Postoperative pathology confirmed moderately to highly differentiated hepatocellular carcinoma (**Figure 1**). Liver radiofrequency ablation was performed on 19 October 2020, for local tumor recurrence. However, unexpectedly, another two lesions were found in the next 2 months. Thus, lenvatinib plus camrelizumab was administered, which was aborted 2 months later because of severe drug-induced liver injury. The patient spent more than 2 months recovering his liver function. The patient was found to have lumbar metastases. Lenvatinib plus camrelizumab was retaken 1 month after liver function returned to normal. Transcatheter arterial chemoembolization (TACE) was performed on 23 April 2021, to inhibit tumor progression in the liver. Stereotactic radiotherapy was adminstered to relieve bone destruction in May. However, the tumor continued to grow in size. Lenvatinib was replaced by sorafenib on 9 August 2021. He underwent TACE on 9 November 2021 and 6 December 2021.

**Figure 2:**
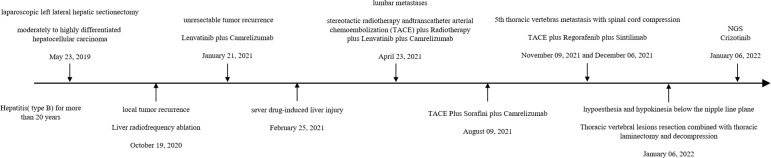
The disease duration of the patient.

The therapy was replaced with regorafenib plus sintilimab after the 5th thoracic vertebral metastasis in November. However, in December 2021, the patient experienced hypoesthesia and hypokinesia below the nipple line plane as 5th thoracic vertebras as metastasis progressed with spinal cord compression. Thoracic vertebral lesion resection combined with thoracic laminectomy and decompression was performed to relieve compression. The pathology of the metastasis is also shown in **Figure 1**, which was slightly different from the primary pathology. A summary of disease duration is shown in **Figure 2**.

Common therapies for unresectable HCC, including tyrosine kinase inhibitors, checkpoint inhibition immunotherapy, radiotherapy, and interventional treatment, seem to have gradually become invalid. The patient suffered a 5th thoracic vertebra metastasis, ending to paralysis from the waist down. Although lumbar decompression was performed to help regain exercise ability immediately, the tumor showed a rapid progression.

Thus, next-generation sequencing (NGS) was used to identify potential molecular-targeted agents. NGS covers all the coding exons, selected introns, and promoters, and analyses all gene mutations, including single-base mutation, DNA intercalation or deletion, gene copy number amplification or loss, and gene fusion or rearrangement. The NGS results of this patient showed MET amplification with copy number variations of 30.2.

**Figure 3:**
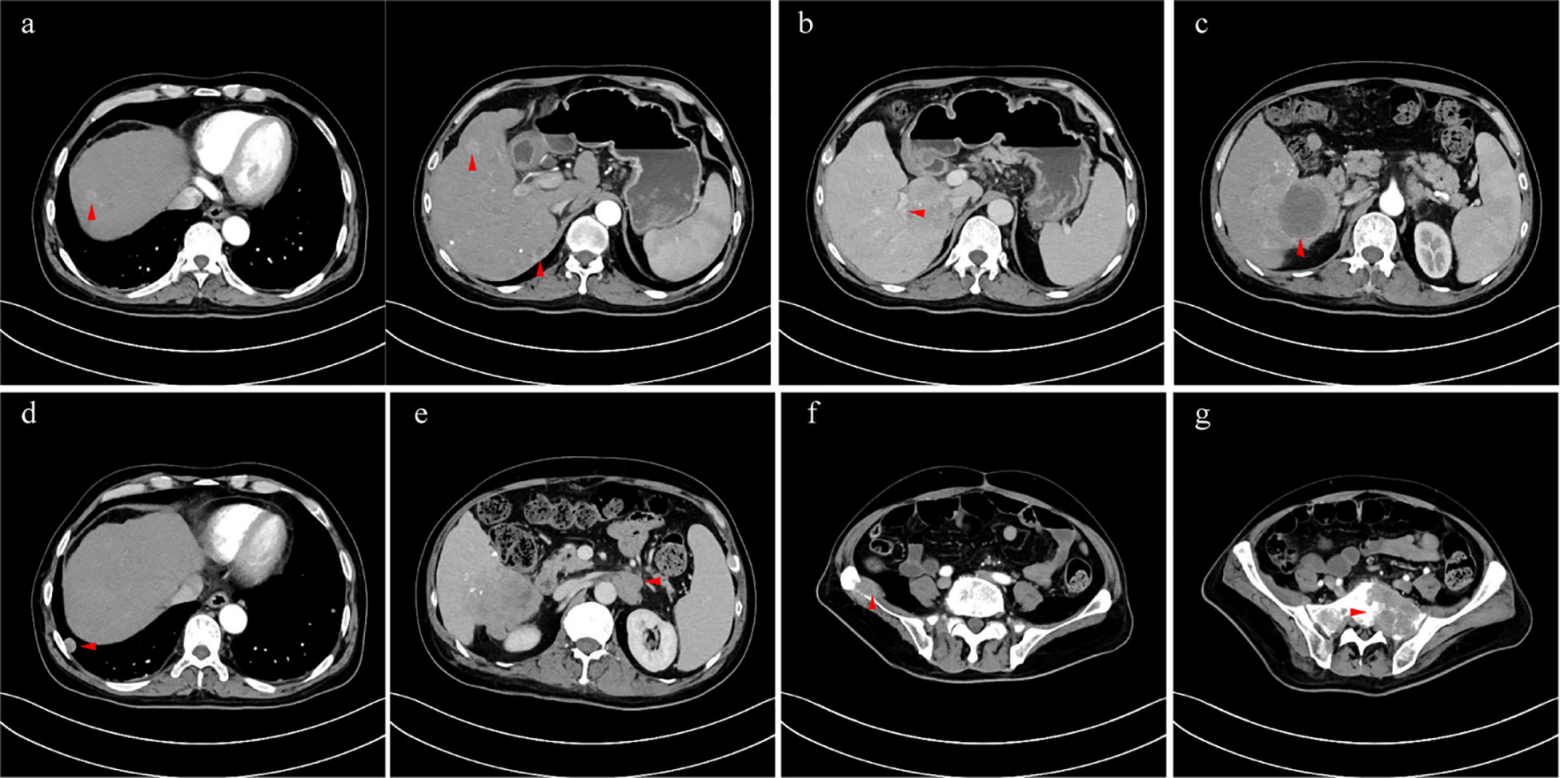
**(A)** intrahepatic multiple lesions (arterial phase); **(B)** tumor invaded the right branch of the portal vein (portal vein phase); **(C)** the hyperintense signal lesion after TACE (arterial phase); **(D)** metastatic tumor in the right lung (arterial phase); **(E)** retroperitoneal metastasis which invaded left renal vein (venous phase); **(F)** metastases of the sacrum (arterial phase); **(G)** metastases of ilium (arterial phase).

**Figure 4:**
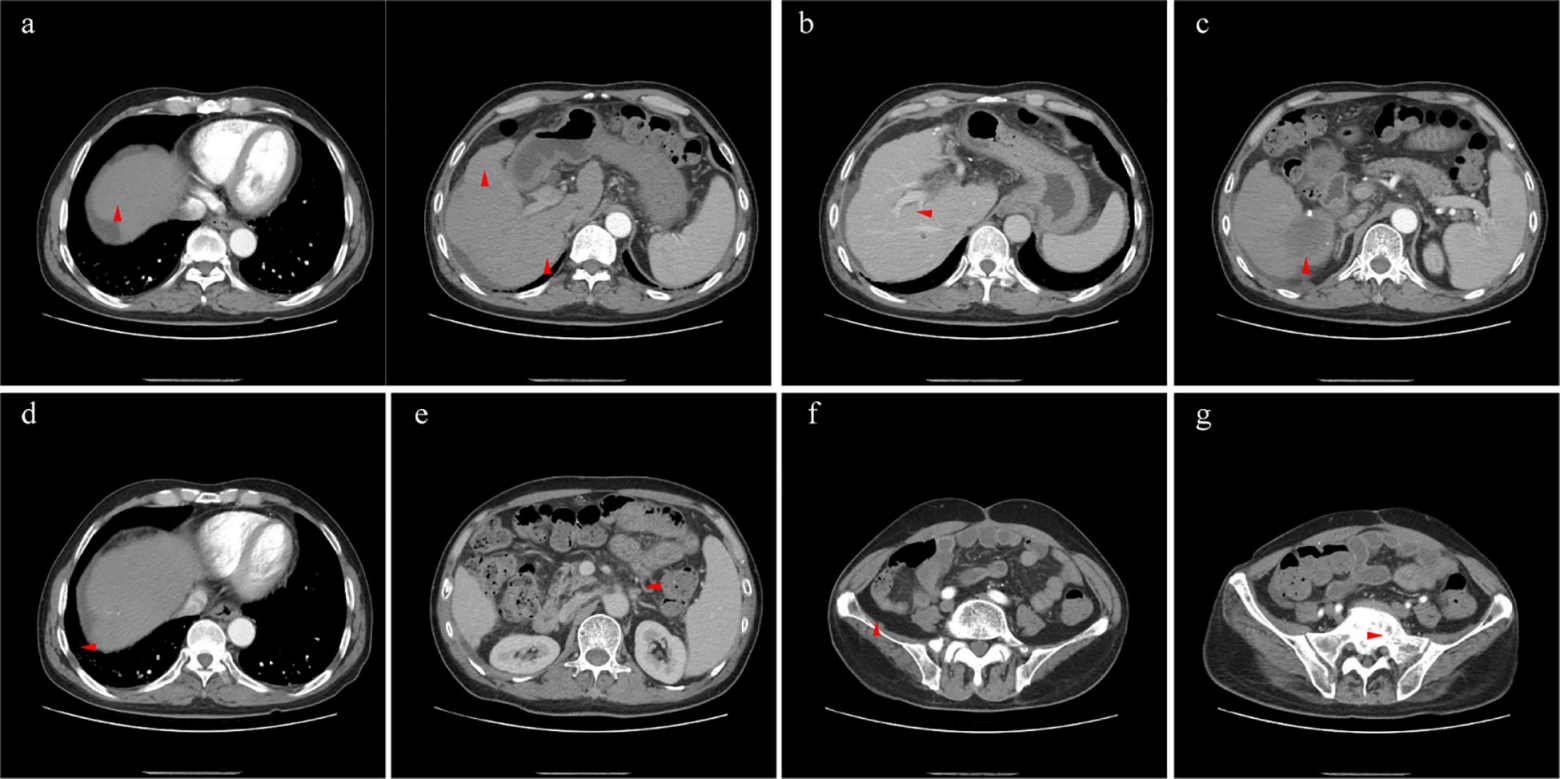
The lesions of liver (**A**, arterial phase), right lung (**D**, arterial phase), retroperitoneal metastasis (**E**, venous phase), sacrum (**F**, arterial phase), and ilium (**G**, arterial phase) shrunk significantly. The right branch of portal vein was released by tumor (**B**, portal vein phase), and necrotic tumor was absorbed generally (**C**, arterial phase).

The patient received crizotinib (200 mg/day) on 24 January 2022 and was continually administered until the last follow-up without any adverse events. The baseline condition and images of the patients are shown in **Figure 3**. The patient presented with mild anemia and acceptable liver damage (40 U/L for ALT and 92 U/L for AST). The AFP level was beyond the detection range and PIVKA-II was approximately 75,000 mAU/ml. Enhanced abdominal CT showed multiple masses in the liver, hilar, retroperitoneal, sacrum, and ilium; right branch of the portal vein invasion. The most recent follow-up visit was on 31 January 2023, and the images (taken on 16 December 2022) are shown in **Figure 4**. The patient achieved excellent tumor remission after targeted treatment. The levels of tumor markers, including AFP and PIVKA-II, declined to the normal range, while routine blood and hepatorenal function tests were stable. The advanced CT scan showed that the diameter of the tumor was observably reduced and even disappeared at several sites.

## Discussion

MET is a proto-oncogene encoding the receptor tyrosine kinase c-MET for hepatocyte growth factor (HGF) (8), and it plays an essential role in tumor onset and progression in different tumor types (9, 10). Once MET is highly activated, it contributes to biogenesis in an autophagy-independent manner through the pathways including the mitogen-activated protein kinase (MAPK), phosphatidylinositol 3-kinase (PI3K), v-srcavian sarcoma (Schmidt-Ruppin A-2) viral oncogene homolog (SRC), and signal transducer and activator of transcription (STAT), leading to cancer progression (11, 12). The MET pathway is altered in several ways including MET overexpression, MET amplification, and MET mutations (13). Although MET overexpression was found in about 27.9% of patients, it is less associated with tumor recurrence or patient survival and MET gene amplification (14, 15). Although rare, MET amplification has been found in various types of tumors, including non-small cell lung cancer (NSCLC) (1%–5%) (16, 17), gastric (6.6%) (18), colorectal (4.4%) (19), and hepatocellular carcinoma (1.7%) (15). It was also reported that 0.69% of AACR GENIE cases presented with MET amplification (20), and many clinical trials have been conducted in recent years, especially for NSCLC, malignant solid tumor, and gastroesophageal adenocarcinoma.

MET amplification may lead to oncogene addiction and confer poor prognosis (10). MET amplification decreases STING levels and antitumor T-cell infiltration, which could result in a weakened IFN response, leading to resistance to immune checkpoint blockade therapy (21). A recent study showed that MET-mediated PARP1 phosphorylation in the nucleus reduced the anti-tumor effects of PARP inhibitors (22). In addition, MET amplification is an important secondary driver in the context of EGFR or other tyrosine kinase inhibitor therapies, leading to drug resistance in many tumors (23–26), such as glioblastoma (27), gastroesophageal adenocarcinoma (28), colorectal cancer (24), and NSCLC (29). However, several studies have found that conventional tumor therapies would be re-sensitive when combined with MET kinase inhibitors (30).

HGF induced by MET was initially identified as a growth factor for hepatocyte and fibroblast-derived cell motility (31). As previously described, MET expression is an independent prognostic factor affecting metastasis and recurrence in patients with small HCC (32). The MET axis promotes glucose uptake, suppresses output, and decreases hepatic glucose production by activating the AMPK-dependent pathway, thereby preventing obesity and insulin resistance (33, 34). The triggered MET pathway upregulates crosstalk between hepatocellular carcinoma and hepatic stellate cells to aggravate cancer (35).

Fluorescence *in situ* hybridization (FISH) and NGS were used to evaluate MET amplification. The absolute gene copy number (GCN) or the ratio of MET to CEP 7 (chromosome 7 probe) was reported through FISH, while NGS compared samples to either a paired normal sample or a standardized set across many genes in a particular NGS assay (13, 36). Although FISH requires less tumor tissue than NGS, it is challenged by significant inter-observer variability and variable results due to tumor sample heterogeneity. The improvement in chromogenic *in situ*-hybridization improves the accuracy of FISH. NGS is limited by the quality of normal samples of non-tumoral DNA from non-neoplastic cells. Thus, it was generally considered that the positive results of NGS was receivable when it should be doubtful for the negative (10, 37, 38).

MET amplification is widely found in different tumors and is a therapeutic target for the treatment of several cancers (39). However, MET inhibitors have vastly different effects in different tumors. In NSCLC, MET receptors such as capmatinib (40), tepotinib (41), and crizotinib (42), have shown substantial antitumor activity in patients with advanced NSCLC. Cabozantinib, an inhibitor of multiple kinases, including VEGFR2 and MET, did affect the metastatic colorectal cancer (43). However, it was difficult to confirm the contribution of blocked MET because of the multiple target spots of cabozantinib. Crizotinib which was approved for the treatment of ALK-positive anaplastic large cell lymphoma, inflammatory myofibroblastic tumors and NSCLC, also showed favorable effects in MET-amplified NSCLC (42). Tivantinib, a small molecular inhibitor of MET, has passed a randomized, double-blind, placebo-controlled, phase 3 study in MET-high hepatocellular carcinoma, which came out with 2.8 month a median progression-free survival and 10.3 months median overall survival (44). However, for glioblastoma, although there were some positive results in *in vitro* and animal experiments, it was still not satisfactory in the clinical practice of MET kinase inhibitor monotherapy (45).

Recently, with the development of NGS, many patients have received effective targeted agents that are not mentioned in conventional treatment guidelines. However, besides MET inhibitors, several agents have different curative effects between tumors, such as IDH1 inhibitor, which showed significant clinical benefit in acute myeloid leukemia but only slight benefit in cholangiocarcinoma (46, 47). This might be due to the different primary gene phenotypes and secondary genetic alterations between tumors.

In conclusion, we report a rare case of advanced HCC treated with a MET inhibitor that achieved excellent tumor remission. Its manifestations confirmed the potential clinical decision support value of NGS and the validity of molecular-targeting treatment.

## Data availability statement

The original contributions presented in the study are included in the article/supplementary material. Further inquiries can be directed to the corresponding author.

## Ethics statement

Written informed consent was obtained from the participant/patient(s) for the publication of this case report.

## Author contributions

YG contributed to the study concept and design, collected, and analyzed the data, and participated in drafting the manuscript. MX contributed to data acquisition and assisted in drafting the manuscript. ZC contributed to data acquisition and critically revised the article. QL conceived the study, participated in study design and coordination, and revised the manuscript critically. All authors read and approved the final manuscript
